# Innovative Water-Insoluble Edible Film Based on Biocatalytic Crosslink of Gelatin Rich in Glutamine

**DOI:** 10.3390/foods9040503

**Published:** 2020-04-16

**Authors:** Yanli Ma, Ruijin Yang, Wei Zhao

**Affiliations:** State Key Laboratory of Food Science and Technology, School of Food Science and Technology, Jiangnan University, 1800 Lihu Avenue, Wuxi 214122, Jiangsu, China; dr_mayanli@163.com (Y.M.); yrj@jiangnan.edu.cn (R.Y.)

**Keywords:** gelatin, glutamine, microbial transglutaminase, crosslink, water-insoluble

## Abstract

Gelatin is a promising candidate for making bioplastic film; however, the water soluble property has limited its applications. Here, we have successfully fabricated a water-insoluble gelatin film with the assistance of biocatalysis. This innovative gelatin film could retain its original shape at ambient temperature (30 °C) or even in boiling water. Type E gelatin could form more covalent crosslinks when compared to that of conventional ones with the same amount of microbial transglutaminase (MTGase), and it exhibits obvious changes in terms of molecular weight, network structure, and mechanical strength. This work could provide a strategy for fabricating water-insoluble gelatin film and open routes for the development of bioplastic film using gelatin.

## 1. Introduction

More than eight-million tons of plastic packaging are used every year, according to The Ellen MacArthur Foundation’s New Plastics Economy Global Commitment Spring 2019 Report [1]. The environmental impact of all this plastic calls for researches related to stopping plastic waste and pollution as well as exploring bioplastic alternatives. Gelatin is a biodegradable polymer that offers opportunity in developing alternatives to petroleum-based plastics for particular applications [2,3]. Being obtained from partial hydrolysis of collagen, gelatin is non-toxic and it has good film forming properties, which make it a potential candidate for packaging [4]. During the film forming process, a physical network of gelatin is formed through the single-strand to triple-helix transition of gelatin chains. The resulting three-dimensional (3D) network stabilized by hydrogen bonds could help gelatin film to insulate oxygen and light. However, this network is thermally reversible and sensitive to water, which are drawbacks for packaging [5,6,7]. 

In contrast to physical network, chemical network formed by covalently linked polymer chains is ‘irreversible’ or ‘permanent’. Chemical network neither disintegrate nor dissolve in aqueous solutions. Crosslinking within the physical network can lead to the development of ‘irreversible’ features. The properties of the resulting network depend on the extent of the crosslinking [8]. Crosslinks have been introduced to create stable net structure for improving ‘permanent‘ properties of gelatin film. Many crosslinkers have been used, such as formaldehyde, glutaraldehyde, carbodiimides, genipin, and transglutaminase [9,10,11,12,13,14]. Among all crosslinking agents, the biocatalytic crosslinker, such as microbial transglutaminase (MTGase), has a distinct advantage over others because of its high efficiency and safety [15]. Being derived from a variant of *Streptoverticillium mobaraense*, MTGase could catalyze the covalent bonding between γ-carboxyamide group of glutamine residues (acyl donors) and ε-amino group of lysine residues (acyl acceptor) [16], resulting in the formation of intra- and inter- molecular crosslinks in gelatin film. Thus, the use of MTGase could be a better alternative for use in the food industry.

Although MTGase has been preferred to modify gelatin film for reducing water solubility, the resulting films were still water soluble at ambient temperature [9,10,11]. To the best of our knowledge, little information regarding the main influence factors in modification has been reported, and producing water-insoluble gelatin film with MTGase modification was still an area not elucidated. In a previous study, we have developed a one-step biocatalysis method to extract type E gelatin and found that the three existing gelatin production methods (the acid process, the liming process, and the enzymatic process) [17,18,19] could produce gelatin with different water solubility under MTGase modification. Furthermore, an innovative water-insoluble gelatin film can be produced while using type E gelatin. Hence, the objective of this study was to analyze the related influence factors in fabricating water-insoluble gelatin film by MTGase modification and evaluate the properties of this innovative gelatin film.

## 2. Materials and Methods 

### 2.1. Materials

Dry and defatted porcine bones and type B gelatin (obtained from alkali process, ~290 Bloom g) were kindly provided by TianZheng Medicine Co., Ltd. (Changchun, Jilin, China), type A gelatin (obtained from acid process, ~300 Bloom g) was purchased from Sigma–Aldrich (Shanghai, China). Pepsin (20000 U/g) was purchased from Sinopharm Chemical Reagent Co., Ltd. (Shanghai, China). Microbial transglutaminase (MTGase, 120 U/g) was obtained from Jiangsu Yi Ming Biological Co., Ltd. (Taizhou, China). The 2, 4, 6-Trinitrobenzenesulfonic acid (TNBS), 5% solution in H_2_O was purchased from Sigma–Aldrich (Shanghai, China). [bis (trifluoroacetoxy) iodo] benzene (BTI) was purchased from Aladdin Reagent Company (Shanghai, China). All chemicals were of analytical grade.

### 2.2. Preparation of Gelatin 

The preparation of type E gelatin was according to our previous method [19]. Briefly, the defatted bone was ground to 30 mesh and then immersed in 1 mol/L hydrochloric acid containing pepsin at 40 U/g bone powder with a solid: solvent ratio of 1:9 (*w*/*v*). The mixture was stirred at 30 °C for 3 h and then centrifuged at 5590 g for 10 min. The precipitate was washed with deionized water at a ratio of 1:2 (*w*/*v*) twice and then collected for gelatin extraction. The aforementioned precipitate was mixed with deionized water at a ratio of 1:2 (*w*/*v*) for gelatin extraction at pH 5.6 for 3 h at 60 °C. The obtained dry gelatin was labeled as type E gelatin and the bloom strength was 295 Bloom g.

### 2.3. Glutamine Analysis

The glutamine content in gelatin was measured according to Kuhn with some modifications [20]. Gelatin solution (1 mL, 0.1 g/mL) was mixed with freshly prepared BTI solution (1mL, 0.4 g/mL in dimethylformamide) and pyridine solution (0.5 mL, 100 μmol/mL in water), subsequently, the mixture was allowed to react at 50 °C for 4h. Subsequently, the water and dimethylformamide were removed completely in a vacuum drier. The precipitate was hydrolyzed by hydrochloric acid (6 mol/L) at 120 °C for 22 h, and the glutamic acid was detected at 338 nm while using a high-performance liquid chromatograph (HPLC, Agilent 1100, Agilent Technologies Inc., Santa Clara, CA, USA). The conditions for HPLC detection were as follows, column: Agilent Hypersil ODS (5 μm, 4.0 × 250 mm); Mobile phase: Eluent A (27.6 mmol/L, pH = 7.2): sodium acetate, triethylamine, and tetrahydrofuran (500:0.11:2.5, V:V:V), Eluent B (80.9 mmol/L, pH = 7.2): sodium acetate, methanol, and acetonitrile (1:2:2, V:V:V); gradient elution mode: 0 min., 8% B; 17 min., 50% B; 20.1 min., 100% B; 24.0 min., 0% B; flow rate: 1.0 mL/min.; and, temperature: 40 °C. Gelatin solution without BTI (only dimethylformamide) was also prepared as control, and the glutamine content was the difference of glutamic acid between the sample and control.

### 2.4. Preparation of Gelatin Films

Gelatin was soaked in deionized water at room temperature (25 °C) for 2 h and then transferred to a water bath at 50 °C to prepare 6.67% (*w*/*v*) gelatin solution. MTGase was dissolved in deionized water to make a stock solution of 8 U/mL, and then added into the gelatin solution to obtain the final concentration of 0, 0.8, and 1.6 U/g gelatin. The samples were prepared and named, as shown in Table 1, E_0_/A_0_/B_0_ were the unmodified samples, while E_1_/E_2_, B_1_/B_2_, and A_1_/A_2_ were the modified ones, the difference between 1 and 2 was the MTGase amount used. Deionized water was used to replace part or all MTGase solution to ensure that all of the samples have the same final gelatin concentration. Afterwards, each sample was stirred gently with a stirrer for 30 min. to obtain homogenous gelatin solution (E_2_ would turn into a gel in excess of 30 min.). Subsequently, the solution was casted on a clean acrylic mold and then maintained in a drying oven at 30 °C for 4 h to form the gelatin film.

### 2.5. Cross-linking Degree

The cross-linking degree of MTGase modified gelatin film was determined as the difference between the number of free amine groups in gelatin before and after modification [21]. The measurement was taken out according to the method of G-Biosciences (Geno Technology Inc., St. Louis, Missouri, USA). Firstly, the gelatin film and TNBS were dissolved in 0.1 M NaHCO_3_ to obtain 100 μg/mL sample solution and 0.01% (*w*/*v*) TNBS solution, respectively. Subsequently, 500 μL sample solution and 250 μL TNBS solution were mixed and incubated at 37 °C for 2 h. Thirdly, 125 μL 1 mol/L HCl and 250 μL 10% SDS were added to the mixture to stop the reaction. A blank sample was prepared in a similar procedure, but 0.1 M NaHCO_3_ solution was used to replace gelatin solution. Finally, the absorbance at 335 nm against the blank was recorded. The crosslinking degree was calculated, as follows:

(1) $$\mathrm{Crosslinking}\;\mathrm{degree}\%=\left(1-\frac{A_{m}}{A_{0}}\right)\times 100\%$$

where A_m_ is the absorbance of the modified sample and A_0_ is the absorbance of the unmodified sample.

### 2.6. Molecular Weight Distribution 

The molecular weight distribution of gelatin film was assessed by sodium dodecyl sulfate polyacrylamide gel electrophoresis (SDS-PAGE), according to the method of Laemmli [22] with slight modifications. All of the samples were mixed with deionized water to the same initial concentration of 2 mg/mL, and then the prepared gelatin solutions were diluted with a sample loading buffer containing 5% (*v*/*v*) β-mercaptoethanol at a ratio of 4:1 (*v*/*v*). The mixtures were immersed in boiling water for 3 min. to denature the proteins. Subsequently, 10 µL of each sample and molecular weight markers (Bio-RaD, Hercules, California, USA) were loaded onto the gel (5% stacking gel and 7.5% resolving gel). Electrophoresis was conducted by a Mini-PROTEAN Tetra Cell (Bio-Rad, USA) with a constant voltage of 80 *V* per gel until the bromophenol blue marker reached the bottom of the resolving gel (about 2 h). The protein bands were fixed and then stained with 0.1% (*w*/*v*) Coomassie Brilliant Blue R-250 in 45% (*v*/*v*) methanol and 10% (*v*/*v*) acetic acid solution for 30 min. and then destained with a solution containing 10% (*v*/*v*) methanol and 10% (*v*/*v*) acetic acid for 48 h. Images of the stained protein bands were captured and analyzed by Image Lab Software (Bio-Rad, USA).

### 2.7. Water Solubility

The water solubility of gelatin film at 30 °C and in boiling water was measured by determining the mass loss of each sample. Gelatin film (20 × 20 × 0.04 mm) was immersed in 30 mL deionized water at 30 °C for 2 h or in 30 mL boiling water for 10 min. The insoluble matter was separated by centrifugation at 5590 g for 10 min. and then dried at 105 ± 2 °C until the weight remained constant (W_2_). The dry weight of similar sized gelatin film was also measured and recorded as W_1_. The water solubility was calculated, as follows:

(2) $$\mathrm{Water}\;\mathrm{solubility}\;\left(\%\right)=\frac{W_{1}-W_{2}}{W_{1}}\times 100\%$$

### 2.8. Mechanical Properties

The tensile tests were conducted by a texture analyzer (TA series, Stable Micro System Co. Ltd., Godalming, UK). The samples (0.04−0.05 mm thick and 15 mm wide) were tested at a gauge length of 25 mm and strain rate of 5 mm min^−1^. All of the samples were previously conditioned at a relative humidity of 50% or 80% for 48 h at 25 °C. The tensile strength was recorded as the stress at sample breakage. The Young’s modulus was calculated from the initial linear region of the stress-strain curve. The toughness was defined as fracture energy and was calculated as the area under the stress-strain curve.

### 2.9. Statistical Analysis

The data were expressed as the mean value ± standard deviation of three replicates. Significant differences between means were calculated by ANOVA in SPSS ver. 19.0 (SPSS, Inc., Chicago, IL, USA). *P* < 0.05 was considered to be statistically significant.

## 3. Results and Discussion

### 3.1. Effect of Glutamine on Crosslinking Degree of Gelatin Films With Mtgase Modification 

For MTGase to have crosslinking activity, the substrate gelatin must have glutamine and lysine. As can be seen from Table 2, type E gelatin, type B gelatin, and type A gelatin possess similar amount of lysine and different amount of glutamine. According to the previous research [14,23], long time liming process hydrolyzes the amide groups of glutamine residues in type B gelatin, therefore large amount of glutamine converts into glutamic acid. However, in our method, type E gelatin undergoes mild preparation process contains greater number of potential crosslinking sits (glutamine). The high amount of glutamine in type A gelatin might be attribute to the raw materials as well as the manufacturing process [24]. This result indicates that all these three types of gelatin could be potentially crosslinked by MTGase in the presence of glutamine and lysine.

**Figure 1:**
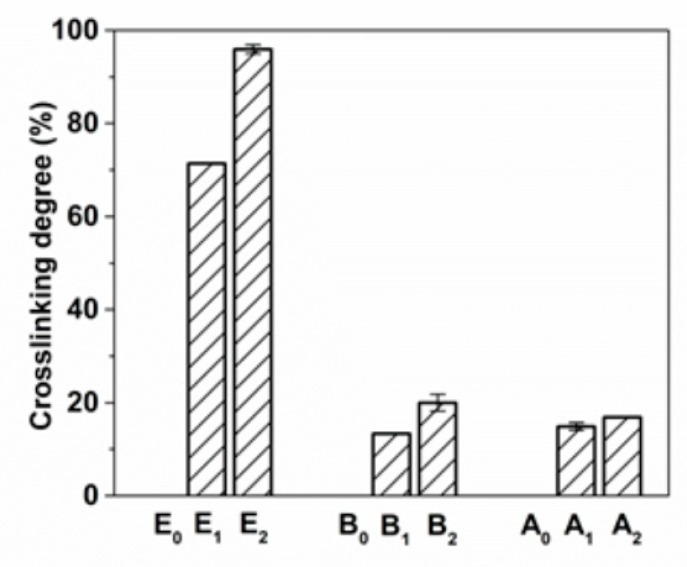
Crosslinking degree of different gelatin films.

All types of gelatin are crosslinked with the addition of MTGase, As can be seen from Figure 1. Type E gelatin exhibits a higher crosslinking degree compared to that of type B gelatin, which might be attributed to the high glutamine content of type E gelatin. However, no high crosslinking degree of type A gelatin modified by MTGase was observed, even though it has the largest amount of glutamine among these three types of gelatin. According to the kinetics of transglutaminase, both acyl donor and acyl acceptor must be in the crevice of the enzyme at the same time in order to make the reaction occur [25]. On the other hand, the conformation of the substrate can also regulate the disposition of crosslinks [26]. Thus, the lower crosslinking degree of type A gelatin might be due to the low chance of γ-carboxyamide group of glutamine residues and ε-amino group of lysine residues to appear simultaneously in the crevice of MTGase [27], as well as the conformation of gelatin chains in type A gelatin, which might limit the enzyme-catalyzed reaction rate. 

### 3.2. Structural Changes in Gelatin Films by Mtgase Modification 

**Figure 2:**
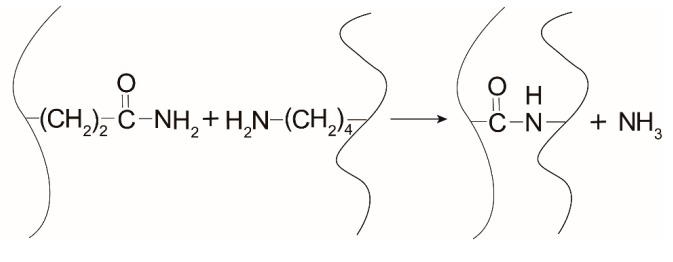
Enzymatic cross-linking of glutamine and lysine residues.

With the addition of MTGase, the reaction within aqueous gelatin solution is shown in Figure 2. Crosslinking that is induced by MTGase can result in different molecular weight distribution compared to that of unmodified gelatin, which is the main basis of the modification. Moreover, new covalent bonds that formed within or between gelatin chains could also result in structural changes in the gelatin film matrix.

**Figure 3:**
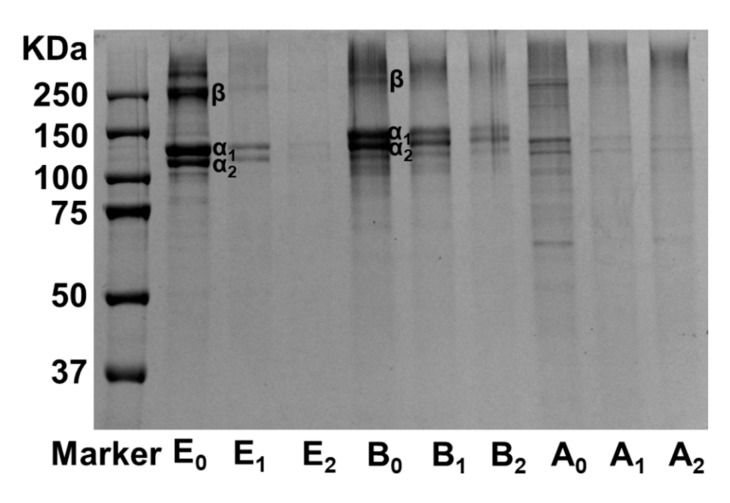
Sodium dodecyl sulfate polyacrylamide gel electrophoresis (SDS-PAGE) patterns of unmodified and MTGase modified type E gelatin, type B gelatin, and type A gelatin films.

In large measure, gelatin properties would depend on molecular weight [16]; therefore, protein pattern changes may confer new character to gelatin film. The molecular weight distribution of gelatin film with and without MTGase was present in Figure 3. As can be seen, α1, α2, and β chains are the major components of unmodified type E gelatin and type B gelatin. Type A gelatin has a broad molecular weight distribution and a higher content of low molecular weight (<100 KDa) fractions. In the addition of MTGase, the band intensity of all components in these three types of gelatin decreased with an increasing concentration of MTGase. This result might be due to the formation of large molecular weight chains in modified gelatin films, indicating the existence of ε-(γ-glutamyl) lysine bonds that are catalyzed by MTGase. Significant difference of molecular weight distribution was observed among modified and unmodified type E gelatin, which was attributed to the higher crosslinking degree when compared to that of the other two types of gelatin. The molecular weight changes of type B gelatin and type A gelatin were not as obvious as that of type E gelatin, also suggesting a strong correlation between crosslinking degree and protein patterns in modified gelatin film.

Generally, structure and character decide the properties and function of certain protein. Although during the extraction process, the triple-helix structure of native collagen was partially broken and random coil was formed, gelatin could be able to recover some native triple-helix structure and realize coil-to-helix transition. This process gives gelatin its unique tertiary structure. However, additional covalent crosslinks could lead to different conformation in gelatin, which X-ray diffraction can measure.

**Figure 4:**
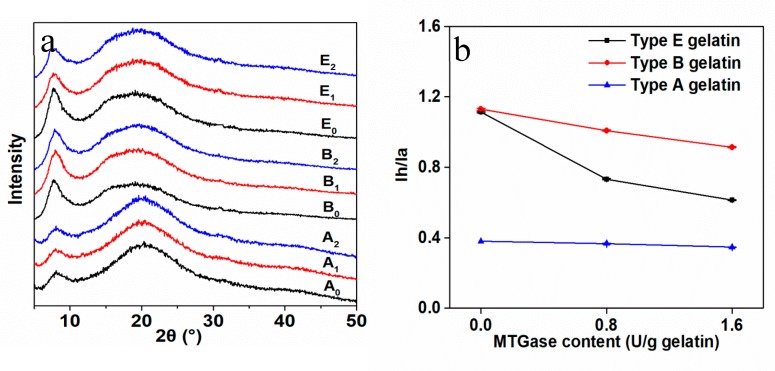
XRD patterns of unmodified and MTGase modified type E gelatin, type B gelatin and type A gelatin films (**a**). Effect of MTGase content on the intensity ratio of triple-helix to amorphous peak (I_h_/I_a_) of unmodified and MTGase modified gelatin films (**b**).

Unmodified samples displayed similar diffraction patterns, a prominent peak at 7° (2θ), which represents the triple-helix configuration, and a broad scattering peak at 20° (2θ), which represents the amorphous fraction, as can be seen from Figure 4a. Slight differences were observed among modified and unmodified type B gelatin and type A gelatin, respectively. Type E gelatin and type B gelatin have a similar peak pattern at 20°, which was different from that of type A gelatin. This phenomenon might be attributed to the different preparing processes and different collagen sources. Usually, the intensity of peak 7° was related to the triple-helix content of gelatin [28,29]. With the addition of MTGase, modified type E gelatin presented a different X-ray diffraction pattern from that of the unmodified, the intensity of peak at 7° was reduced as the amount of MTGase increased. This suggested that the crosslinks that are introduced by MTGase possibly prevent the formation of triple-helix [30]. 

Figure 4b gives a clear description on structural changes of three types of gelatin with and without MTGase. The intensity ratio of the triple-helix to amorphous peak (I_h_/I_a_) significantly decreased in type E gelatin as the MTGase content increased, and a few changes were observed in type B gelatin. For type A gelatin, the intensity ratio (I_h_/I_a_) was lower than that of type E gelatin and type B gelatin, which might due to the broad molecular weight distribution of type A gelatin, and whatever the addition amount of MTGase, the intensity ratio (I_h_/I_a_) was almost the same value. All of these results suggested that I_h_/I_a_ was associated as the crosslinking degree of modified gelatin films, which decreased with the crosslinking degree increased. 

### 3.3. Water Solubility and Mechanical Properties of Gelatin Films with the Modification of Mtgase

Gelatin could be used as a packaging material and biomaterial because of its good biocompatibility and degradability. Usually, some of its original characters should be improved in order to meet the requirement of particular use, such as its excessive water solubility or sometimes poor mechanical properties. Here, two obvious changes in gelatin film with the modification of MTGase were listed.

**Figure 5:**
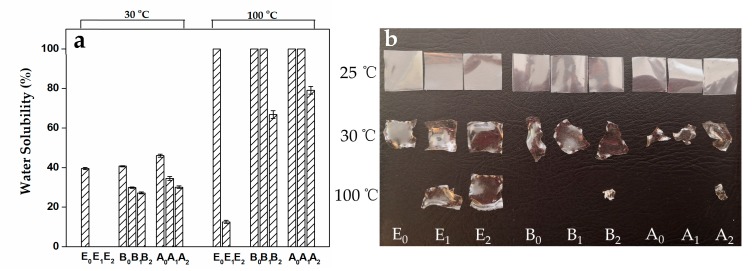
Water solubility (at 30 °C and 100 °C) of unmodified and MTGase modified gelatin films (**a**) and their photographs before and after water solubility test (**b**).

Figure 5a shows the water solubility of modified and unmodified gelatin film at ambient temperature (30 °C) and boiling water (100 °C). All types of gelatin film without MTGase modification were partially dissolved in water at 30 °C which was about the melting temperature of gelatin (type E gelatin, 31.0 °C; type B gelatin, 30.8 °C; type A gelatin, 28.9 °C). With the addition of MTGase, the type E gelatin film exhibited different solubility compared to that of the other two types of gelatin film. At 100 °C, only E_2_ was completely insoluble, and the other eight gelatin films exhibited various water solubility, indicating that the water-insoluble gelatin film could be fabricated while using type E gelatin with the assistance of MTGase.

Generally, gelatin film is predominantly formed by hydrogen bonds which are disrupted on heating; therefore, normal gelatin film is thermally reversible [31]. However, additional covalent bonds that are introduced by MTGase could “fix” the gelatin network and restrict the gelatin chain flexibility, resulting in low water solubility [32]. The higher the crosslinking degree, the lower the dissolution of the modified gelatin film, as can be seen from Figure 5. Furthermore, we have found that the insoluble part of B_2_ and A_2_ transformed into a little ball in boiling water, while E_2_ and E_1_ kept their original shapes. This phenomenon could be attributed to the enough covalent bonds that are induced by MTGase, which are strong enough to maintain the integrity of the films. 

The mechanical properties of gelatin film after conditioning under a high relative humidity (80%) were compared with that in a relative humidity of 50%. For each type of gelatin, the tensile strength, modulus, and toughness increased with increasing crosslinking degree at both humidity conditions, as can be seen from Table 3. Crosslinks could strengthen the interaction between gelatin chains, leading to a higher mechanical strength. However, higher relative humidity (80%) conditioning greatly decreases the mechanical strengths of all samples, which should be attributed to the increased water absorption in a higher humidity condition. Higher water content will increase the distance between gelatin chains, thus reducing the number of polymer chains per unit volume, leading to a lower mechanical strength [33]. When compared to type B gelatin film and type A gelatin film, Type E gelatin film exhibited relatively high tensile strength and toughness at both conditions, which suggests the large potential of modified type E gelatin film for bioplastic applications. 

## 4. Conclusions

In summary, MTGase modification can help to fabricate water-insoluble gelatin film. The amount of glutamine and molecular weight distribution of gelatin may have influence on the efficiency of biocatalytic crosslinking. An innovative boiling-water-insoluble gelatin film could be fabricated with the assistance of MTGase while using type E gelatin produced by the enzymatic method. The water-insoluble gelatin film has high molecular weight polymer chains, stable network, and relatively high mechanical strength. This study, focusing on the factors that influence MTGase modification of gelatin, could provide a strategy for fabricating water-insoluble gelatin film for particular uses.

## Figures and Tables

**Table 1:** Name and MTGase concentration of 9 gelatin samples.

Sample	Name		
Type E gelatin	E_0_	E_1_	E_2_
Type B gelatin	B_0_	B_1_	B_2_
Type A gelatin	A_0_	A_1_	A_2_
MTGase concentration (U/g gelatin)	0	0.8	1.6

**Table 2:** Glutamine and lysine content (mmol/100g) in type E gelatin, type B gelatin, and type A gelatin.

Amino Acid	Type E Gelatin	Type B Gelatin	Type A Gelatin
Glutamine	22.33 ± 1.06	13.83 ± 0.35	27.52 ± 0.79
Lysine	21.37 ± 0.00	21.47 ± 0.33	21.41 ± 0.06

**Table 3:** Mechanical properties of gelatin films under different conditions.

Sample	RH 50%				RH 80%			
	Youngs Modulus (MPa)	Tensile Strength (Mpa)	Elongation at Break (%)	Toughness (MJm^−3^)	Youngs Modulus (Mpa)	Tensile Strength (Mpa)	Elongation at Break (%)	Toughness (MJm^−3^)
E_0_	1796.64 ± 20.13 ^d^	88.48 ± 1.52 ^c^	6.92 ± 0.14 ^c^	3.15 ± 0.10 ^e^	1.02 ± 0.04 ^e^	1.34 ± 0.04 ^ef^	141.99 ± 7.10 ^de^	0.76 ± 0.03 ^g^
E_1_	2007.78 ± 25.11 ^b^	92.25 ± 1.02 ^b^	7.29 ± 0.21 ^b^	4.08 ± 0.11 ^b^	1.34 ± 0.05 ^b^	2.23 ± 0.07 ^b^	172.01 ± 5.60 ^b^	1.37 ± 0.02 ^c^
E_2_	2090.86 ± 19.23 ^a^	96.83 ± 1.23 ^a^	7.65 ± 0.18 ^a^	5.40 ± 0.17 ^a^	1.64 ± 0.06 ^a^	3.64 ± 0.09 ^a^	186.97 ± 8.34 ^a^	2.19 ± 0.04 ^a^
B_0_	1811.07 ± 15.18 ^d^	84.32 ± 2.01 ^d^	6.90 ± 0.14 ^c^	3.46 ± 0.12 ^d^	0.96 ± 0.03 ^ef^	1.27 ± 0.04 ^f^	136.29 ± 4.81 ^e^	0.87 ± 0.03 ^f^
B_1_	1843.25 ± 20.01 ^c^	89.56 ± 1.54 ^c^	7.21 ± 0.09 ^b^	3.73 ± 0.09 ^c^	1.12 ± 0.04 ^d^	1.41 ± 0.03 ^eg^	154.45 ± 5.72 ^c^	1.11 ± 0.02 ^d^
B_2_	1868.23 ± 15.26 ^c^	94.09 ± 1.16 ^b^	7.42 ± 0.11 ^ab^	3.84 ± 0.16 ^c^	1.22 ± 0.06 ^c^	1.94 ± 0.07 ^c^	177.65 ± 6.88 ^ab^	1.52 ± 0.02 ^b^
A_0_	1609.35 ± 19.16 ^f^	71.06 ± 1.32 ^f^	4.21 ± 0.19 ^f^	1.62 ± 0.07 ^g^	0.85 ± 0.03 ^g^	1.22 ± 0.03 ^g^	88.13 ± 2.41 ^g^	0.58 ± 0.01 ^h^
A_1_	1655.57 ± 15.42 ^e^	72.63 ± 0.99 ^f^	4.61 ± 0.08 ^e^	1.66 ± 0.05 ^g^	0.85 ± 0.02 ^g^	1.59 ± 0.05 ^d^	115.85 ± 3.79 ^f^	0.99 ± 0.02 ^e^
A_2_	1668.75 ± 10.39 ^e^	78.04 ± 1.28 ^e^	5.73 ± 0.11 ^d^	2.03 ± 0.06 ^f^	0.93 ± 0.01 ^f^	1.61 ± 0.02 ^d^	118.09 ± 2.90 ^f^	0.95 ± 0.01 ^e^

Different lowercase letters show significant differences (*P* < 0.05) among the same row.
